# Changes to Soil Microbiome Resulting from Synergetic Effects of Fungistatic Compounds Pyrimethanil and Fluopyram in Lowbush Blueberry Agriculture, with Nine Fungicide Products Tested

**DOI:** 10.3390/microorganisms11020410

**Published:** 2023-02-06

**Authors:** Austin W. Lloyd, David Percival, Morgan G. I. Langille, Svetlana N. Yurgel

**Affiliations:** 1 Department of Plant, Food, and Environmental Sciences, Dalhousie University, Truro, NS B2N 5E3, Canada; 2 Department of Pharmacology, Dalhousie University, Halifax, NS B2N 5E3, Canada; 3 USDA, ARS, Grain Legume Genetics and Physiology Research Unit, Prosser, WA 99350, USA

**Keywords:** *Vaccinium angustifolium*, *Vaccinium myrtilloides*, fungicides, pyrimethanil, fluopyram, soil microbiome, metagenomics

## Abstract

Lowbush blueberries (*Vaccinium* spp.) are a crop of economic significance to Atlantic Canada, Quebec, and Maine. The fruit is produced by the management of naturally occurring plant populations. The plants have an intimate relationship with the soil microbiome and depend on it for their health and productivity. Fungicides are an important tool in combatting disease pressure but pose a potential risk to soil health. In this study, amplicon sequencing was used to determine the effects of six fungistatic compounds both alone and in combination via nine commercially available fungicide products on the bacterial and fungal microbiomes associated with lowbush blueberries and to study whether these effects are reflected in crop outcomes and plant phenotypes. One fungicide, Luna Tranquility, a combination of fluopyram and pyrimethanil, was found to impart significant effects to fungal and bacterial community structure, fungal taxonomic abundances, and bacterial functions relative to control. The two fungicides which contained fluopyram and pyrimethanil as single ingredients (Velum Prime and Scala, respectively) did not induce significant changes in any of these regards. These results suggest the possibility that these microbiome changes are the result of the synergistic effect of fluopyram and pyrimethanil on soil microbiomes. While these results suggest a significant disruption to the soil microbiome, no corresponding changes to crop development and outcomes were noted. Ultimately, the majority of the fungicides analysed in this trial did not produce significant changes to the soil microbiome relative to the untreated group (UTG). However, one of the fungicide treatments, Luna Tranquility, did produce significant changes to the soil ecosystem that could have longer-term effects on soil health and its future use may merit additional investigation onto its ecotoxicological properties.

## 1. Introduction

Mutualistic relationships between plants and microorganisms are an essential part of the agricultural system, with these interactions being necessary to ensure that the plant will thrive in its environment [1]. These symbioses are particularly relevant for lowbush blueberries (*Vaccinium angustifolium* and *Vaccinium myrtilloides*), a crop which is produced through managing wild plant populations throughout eastern Canada and Maine. As is the case with many species in the order Ericales, lowbush blueberries form ericoid mycorrhizae (ErM)—a specialized form of fungal symbiont—which allow the plants to gain more direct access to forms of nitrogen which would otherwise be locked up in organic matter, thus allowing the plants to survive in nutrient-poor soils [2,3,4,5]. 

The identities of the fungi which comprise the ErM fungi are fraught with uncertainty. It has been established that the group contains both Ascomycetes and Basidiomycetes (Martino et al. 2018). *Rhizoscyphus ericae* (formerly Hymenoscyphus ericae), is well-known for its symbiotic properties, and is one of the best studied taxa [3,6]. However, many other fungal taxa have been identified as potential ErM, such as species within Helotiales that have been associated with higher nutrient content in the leaves of *Vaccinium angustifolium* [7]. Basidiomycota species such as members of Sebacinales have been established to form ErM, and other Basidiomycota species such as some of the genus *Clavaria* have been identified as potentially doing so [4,8]. In the bacterial realm, a study has shown Bradyrhizobium to be the genus with the greatest relative abundance overall, one which has been found to correlate with a higher concentration of nitrogen in plant leaves [7]. In general, associations have been made between higher microbial abundances with higher eukaryotic taxonomic richness and increased soil fertility [9]. And, managed soils have been demonstrated to have an increase in factors potentially deleterious to crop success such as an increase in pathogen abundance and a decrease in arbuscular mycorrhizae [9]. With these factors in mind, it is thus important to monitor the effect of management practices on the microbiome in order to maintain soil health. 

Fungicides are a critical component of lowbush blueberry production. The question of the effects of pesticides on the health of the soil microbiome is one of considerable environmental concern. However, given the multiplicity of pesticides, both in terms of target organism (insecticide, fungicide, herbicide, etc.) and active ingredient mode of action, mobility, and persistence, their effects on the soil microbiome can be difficult to predict. Previous research has suggested that the interactions between fungicides and the soil microbiome in lowbush blueberries production system merits additional investigation. We previously reported that the diversity and structure of fungal communities in soils treated with prothioconazole and chlorothalonil differed significantly from that in untreated soil plots [10]. Additionally, a fungal family, which represented potential blueberry symbionts was significantly increased in its relative abundance in plots treated with prothioconazole relative to untreated control plots. Significant changes in bacterial function in response to the fungicide treatments, which was reflected in the increase in degradation of either halocarbons or other synthetic compounds was also reported [10]. While these changes to the microbiome have been noted with regards to fungicide application, it was not clear whether these microbial changes would have significant impacts on the crop itself. 

In this study we broadened the number of fungicides applications to establish a more complete understanding of the interactions between fungicides and soil ecology. Nine commercially available products comprising six active ingredients and four mechanisms of action formed the core of this trial. This array of trial products allows for assessment of a wider range of active ingredients, to broaden the knowledge base set forth in previous research, and to explore some interactive effects of the two fungicides. Additionally, data regarding plant development and phenotypes taken from the trial were integrated into the analyses to investigate whether there are correlations between microbiome changes and the plants themselves in this context. It was hypothesized that some of the fungicides in use might lead to significant changes to the soil microbiome, that these changes would differ from one active ingredient to the next, and that these microbiome effects might lead to changes in the phenotype of the plants themselves. 

## 2. Materials and Methods

### 2.1. Site and Management

The fungicide trial was conducted at a commercial blueberry production field in Debert, Nova Scotia (45.438321, −63.453072) which was in the harvest year of the cropping cycle. Each plot consisted of a 4 m × 4 m area separated from its neighbor plots by a 2 m buffer zone. The experimental design consisted of nine fungicide treatments (Table 1), and one untreated control (UTG) across five replications, arranged in a randomized complete block design. Fungicides were applied on 26 June 2020, and on 8 July 2020. Soil samples were taken on the 20 July 2020. All fungicides were applied at rates at or below the manufacturers’ specified rates, with the exception of Miravis Bold and Scholar which are not currently in use for lowbush blueberries. 

### 2.2. StemPlant Analyses

From each plot in four of the reps, 15 stems were collected on 8 July. The length of each stem was measured, and the number of both floral and vegetative nodes was counted. Additionally, the vegetative and floral development stage of each stem was noted. Furthermore, the incidence and severity of *Monilinia* and *Botrytis* on both vegetative and floral nodes were taken into account by noting both the number of infected nodes, as well as the severity of each infected node, which was estimated visually. Finally, the presence of *Septoria*, was taken into account for each stem. In 15 August, shortly before harvest, 15 stems samples were taken from each plot. For each stem, the number of ripe berries was counted, as well as the number of berries which were underdeveloped or otherwise unmarketable. As with the first round of sampling, the length of each stem was taken into account as well. 

### 2.3. Soil Sampling, DNA Isolation, and Sequencing

From each plot, three soil samples were taken at random from the top 10 cm of soil, leading to a total of 150 soil samples. Soil samples were immediately placed on ice in the field and were sifted through a 2 mm sieve before being transferred into a −80 °C freezer. For each sample, 0.250 g (wet weight) of sifted soil was used for DNA isolation, which was performed using the Omega Biotek E.Z.N.A. Soil DNA extraction kit (Omega Bio-tek, Inc., Norcross, GA, USA) as per manufacturer specifications. DNA sequencing was performed on Illumina MiSeq at the Dalhousie University IMR using the Microbiome Helper protocol [11]. 

### 2.4. Sequence Processing and Data Analysis 

The DNA sequences which had been derived from soil samples were processed using QIIME2 [11]. The sequences were trimmed of their primers with Cutadapt [12]. The paired-end forward and reverse reads were stitched using PEAR [13]. Low-quality sequences were removed using QIIME2’s q-score-joined function. Sequences were organized into amplicon sequence variants (ASV) with QIIME2’s Deblur plug-in [11,14,15]. ASV which comprised less than 0.1% of the total sequences were removed to reduce the prevalence of bleed through error, which Illumina estimates to be around 0.1% [11]. Taxonomic classifications were assigned referencing SILVA databases (16S rRNA V6-V8, and fungi-specific ITS2) using the naïve-Bayes scikit-learn function in QIIME2 [16,17]. ASV which were unassigned at the division level were filtered from the dataset, given the high probability that they were chimeric sequences. Additionally, mitochondrial and chloroplast sequences were removed [11]. From the remaining ASV, two tables of ASV counts were constructed per sample—for the 16S rRNA and ITS2 datasets, respectively—along with a list of present taxonomic identities, and a phylogenetic tree [11]. For alpha and beta diversity analyses, the datasets were rarefied to the depth of the minimum acceptable read size (4278 reads for ITS2, and 5089 reads for 16S rRNA). Alpha diversity metrics (species richness (Chao1 index) and Simpson’s evenness index) were calculated using QIIME2 compared using the Wilcoxon test. UniFrac matrices were also generated using QIIME2, and their variance was compared using the ADONIS test [18]. Functional potentials were predicted using PICRUSt2 [19]. Differential relative abundances for both 16S rRNA and ITS2 and functional pathways were determined using ALDEx2, with pairings which demonstrated significant differences in beta-diversity being tested for significant differences in taxonomic abundances [20]. For the comparison of the relative abundances an unrarefied dataset was used. All samples which fell below the minimum read count were discarded for all the ALDEx2 analyses. In addition to comparing each fungicide individually to UTG, all of the fungicide treated plots were also analysed as a whole, combined treatment group (CTG), to determine whether the presence of any fungicide had significant effect, regardless of the compound. All data visualizations were produced using ggplot2 [21]. For the stem samples, each treatment group, each factor analysed was normalized through rarefaction to the number of samples present in the treatment group with the fewest data points. To determine whether variance in each of these factors was significantly correlated to each fungicide treatment, an ANOVA test was conducted, followed by Fisher’s LSD test to determine which treatments yielded results which differed significantly from each other [22]. 

## 3. Results

### 3.1. Phenotype Expression, Crop Outcomes, and Disease Incidence

Fungicide treatments had a significant effect on the rate of floral bud development (*p* < 0.05), with Miravis Prime having the least developed floral buds at sample time, and also being the only treatment, which differed significantly from UTG (Table 2). However, the number of floral nodes, as well as the number of vegetative nodes did not differ significantly with treatment. The prevalence and severity of *Monilinia* on vegetative nodes did not vary significantly between treatment groups (*p* > 0.05) and the pathogen was not observed on any of the floral nodes sampled. *Botryris* was found on the floral nodes of only one of the 539 stem samples analysed and was not observed on any of the vegetative nodes. 

### 3.2. Data Characteristics

Once contaminants were removed from the bacterial dataset, 9635 features were present across 153 samples, with a mean reads/sample of 18,949 16S rRNA reads/sample. With the 20 failed samples excluded from the dataset, the total number of features was reduced to 9631 and the mean reads/sample count was increased to 21,249. The majority of all reads corresponded to Proteobacteria (34% of total 16S rRNA reads), Actinobacteria (30% of total 16S rRNA reads), and Acidobacteria (19% of total 16S rRNA reads).

The fungal dataset, once contaminant sequences were removed, contained a total of 2018 features, with a mean frequency of 15,753 ITS reads/sample, with a total of 153 samples. The exclusion of failed samples lowered the total number of identified features to 1994 and raised the mean reads/sample to 18,251. Ascomycota and Basidiomycota were the most well-represented fungal phyla, together comprising more than approximately 75% of ITS reads in each sample.

### 3.3. Alpha Diversity

**Figure 1:**
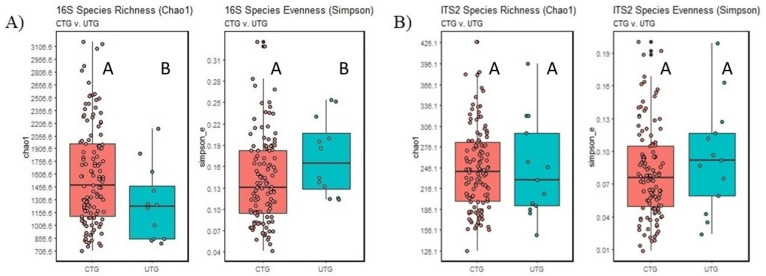
Alpha diversity metrics for CTG and UTG. (**A**): Bacterial community. (**B**): Fungal community. For each metric, columns marked by differing letters are significantly different (Wilcoxon test, BH-adjusted *p* < 0.05).

In the bacterial and fungal datasets, species richness (Chao1 index) and Simpson’s evenness index did not vary significantly as a result of the individual treatment groups relative to UTG. However, it did show that there were significant differences in both species richness and evenness between UTG and CTG, with CTG exhibiting a higher species richness and a lower species evenness (Wilcoxon test, BH-adjusted *p* < 0.05) than UTG (Figure 1A). In contrast with the bacterial dataset results, no significant variance in fungal species richness and evenness could be attributed to fungicide application in comparing CTG to UTG (Wilcoxon test, BH-adjusted *p* > 0.05) (Figure 1B).

### 3.4. Beta Diversity

**Figure 2:**
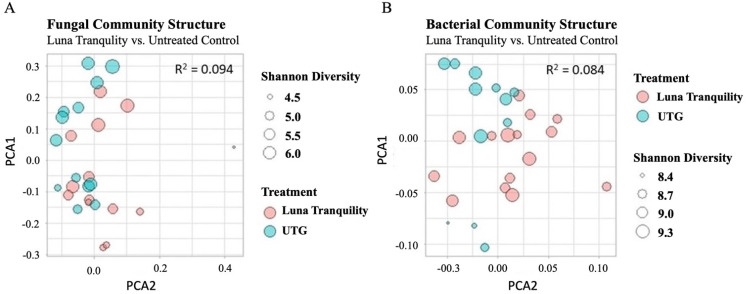
PCoA of community structures, Luna Tranquility and UTG. PCoA comparing Luna Tranquility (LT) and Untreated Control (UTG). (**A**)—fungal; (**B**)—bacterial populations. (ADONIS test, *p* < 0.05). See supplemental Figures S1 and S2 for other treatments.

ADONIS tests comparing the significance of difference between UniFrac matrices for each treatment group found that 10% of variance in the 16S rRNA dataset could be attributed to the effect of individual treatments (*p* < 0.05). Notably, Luna Tranquility was the only treatment group responsible for significant variance from UTG, causing 9% of the overall variance between the two groups (Figure 2). With the fungal ITS2 dataset, Luna Tranquility was also the only treatment group which differed significantly different from control (9% of variance, *p* < 0.05) (Figure 2). While Luna Tranquility, a fungicide with two active ingredients (fluopyram and pyrimethanil) elicited significant differences relative to UTG in both the fungal and bacterial datasets, neither Velum Prime (a.i. fluopyram), nor Scala (a.i. pyrimethanil) caused significant changes relative to UTG. In fact, in the fungal dataset, the largest difference in community structures was found between Luna Tranquility and Scala, with treatment being found accountable for 15% of variance. For both the 16S rRNA and ITS2 datasets, no significant differences were found in the community structure of CTG and UTG. 

### 3.5. Effect of Fungicides Application on Soil Community Structure

**Figure 3:**
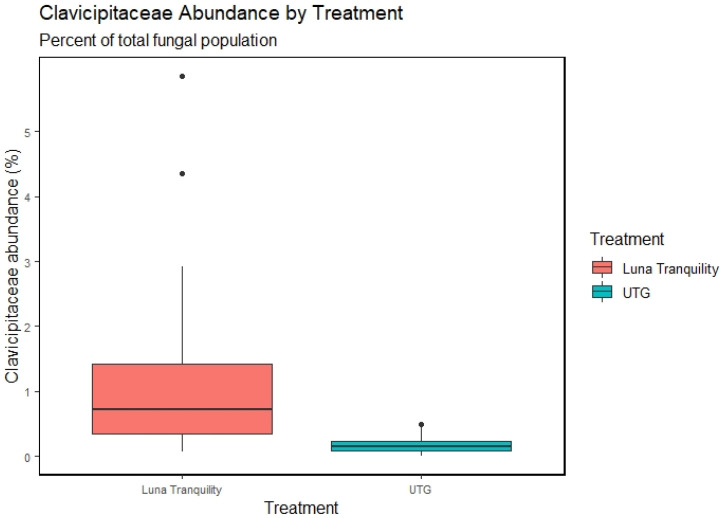
Clavicipitaceae abundances by treatment group. Abundances of Clavicipitaceae were significantly increased in plots treated with Luna Tranquility relative to UTG (Wilcoxon test, BH-corrected *p* value < 0.05).

No bacterial taxa were found to differ significantly in relative abundance between UTG and CTG and none of the individual fungicide treatment groups were associated with significant changes relative to UTG. Comparing the treatments in a pairwise manner found numerous taxa which differed significantly in relative abundance. In accordance with the significant variance between UniFrac matrices, these differential relative abundances centered around Luna Tranquility. One family, Clavicipitaceae was found to differ significantly between Luna Tranquility and control (UTG), with a significantly increased relative abundance in Luna Tranquility relative to UTG (Figure 3). 

### 3.6. Effect of Fungicides Application on Bacterial Functional Potentials

**Figure 4:**
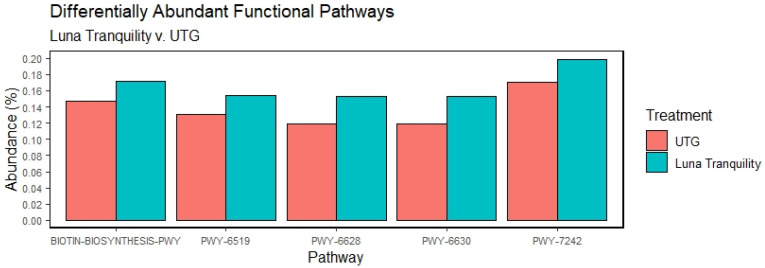
Functional pathways which differed significantly between Luna Tranquility and UTG. Mean abundances of functional pathways which differed significantly (Wilcoxon test, BH-adjusted *p* < 0.1) between Luna Tranquility and UTG. Only those pathways with greater than 0.1% abundance shown. Pathways shown above: BIOTIN-BIOSYNTHESIS-PWY—biotin biosynthesis I; PWY-6519—8-amino-7-oxononanoate biosynthesis I; PWY-6628—superpathway of L-phenylalanine biosynthesis; PWY-6630—superpathway of L-tyrosine biosynthesis; PWY-7242—D-fructuronate degradation.

Significant differences in the relative abundances of functional pathways were found between Luna Tranquility and UTG, with 22 pathways having significantly different relative abundances between the two groups. The pathways with a mean abundance of greater than 0.1% are shown in Figure 4. The most abundant of these pathways was PWY-7242, through which D-fructuronate is degraded, and exhibited a higher relative abundance in the Luna Tranquility treatment group than in UTG. Additionally, BIOTIN-BIOSYNTHESIS-PWY—biotin biosynthesis I—through which the microbe creates biotin, was found to be of increased relative abundance in Luna Tranquility. Additionally, PWY-6519 (8-amino-7-oxononanoate biosynthesis I), PWY-6628 (superpathway of L-phenylalanine biosynthesis), and PWY-6630 (superpathway of L-tyrosine biosynthesis) all presented higher relative abundance in Luna Tranquility than in UTG. No other fungicide treatment provoked significant changes in relative abundances of functional pathways relative to UTG. 

## 4. Discussion

In our previous study of the relationship between fungicides and soil health in wild blueberries production system, we found that whether a soil was treated or untreated was the primary catalyst of significant soil changes, as opposed to either fungicide individually [10]. It this study we substantially extended the set of tested compounds with fungicidal activity and identified one fungicide, Luna Tranquility, which had a significant effect on soil bacterial microbiome. For example, only communities treated with Luna Tranquility were visually separated from untreated control based on PCoA analysis (Figure 2). Both fluopyram and pyrimethanil, the active ingredients in Luna Tranquility, have been associated with community-level changes to soil microbial populations in previous research. One study, found that treatment with fluopyram led to significant changes in the overall community structure (both bacteria and fungi combined) [23], while another study showed that bacterial community-level changes corresponding to pyrimethanil treatment were affect by interactions between pyrimethanil dosages and rainfall rates [24]. However, in our study we did not detect a significant effect of separate applications of Scala (pyrimethanil) or Velum Prime (fluopyram) on soil microbiome. Our data also suggested that the changes in the soil microbiome, apparently triggered by Luna Tranquility, are the result of the synergetic effect of its two active ingredients. 

### 4.1. Effects of Fungicides on Taxonomic Profiles

We did not detect any significant changes in fungal species richness or evenness as a result of fungicide treatment, while bacterial richness was increased, and evenness decreased with fungicides application. Similarly, pyrimethanil has been shown to lead to a temporary increase in bacterial diversity [24]. On the other hand, our previous research showed a significant effect of fungicide application on fungal alpha diversity but not on bacterial [10]. These differences suggest a complex interaction between microbiomes and fungicides in soils, which might be further affected by environmental factors including rainfall, soil composition, and temperature. This point is corroborated by Ng et al. who found that changes to bacterial richness under Pyrimethanil differed depending on the amount of rain that the soil had received [24]. Additionally, the temporary nature of the changes to richness noted in that paper may confound our ability to draw conclusions about the effects of fungicides on microbial alpha diversity under field conditions, without the ability to compare taxonomic profiles taken from multiple timepoints [24]. Additionally, it is notable that in our 2020 trial, samples were taken twelve days after the final fungicide application, while a month had elapsed between spraying and sampling in the trial that we conducted in 2019 [10].

No bacterial taxa differed significantly between untreated soils and soils treated with any fungicide tested in this study. These results are consistent with our previous findings, in which no bacterial taxa were identified as differentially represented between fungicides treated and untreated soils [10]. However, there were several other reports pointing on the effect of fungicides on bacterial abundances. Pyraclostrobin, the sole active ingredient in Cabrio and one of two in Merivon, has been associated with significant changes in bacterial taxa in fluvio-aquic soils [25]. Additionally, Fluxapyroxad, found in Sercadis as well as Merivon, reduced the ratio of Gram-positive to Gram-negative bacteria based on phospholipid fatty acid profiles [26]. However, these studies were conducted in laboratory conditions in which fungicides were applied directly to the soil, suggesting that foliar applications of these compounds, as well as a number of environmental factors may significantly mitigate the effects of the fungicide on the soil community. On the other hand, one fungal family, Clavicipitaceae, was significantly increased in Luna Tranquility treated soils compared to UTG. A genus from this family, *Metarhizium*, has been identified as both a root endophyte associated with promoting root growth, and an insect pathogen which has been studied for use as a biopesticide [27,28]. These findings suggested that combination of fluopyram and pyrimethanil as active ingredients might have a side benefit to the crop of increasing root symbiosis. 

### 4.2. Fungicides and Functional Potential

Luna Tranquility induced significant differences in relative abundances of MetaCYC pathways compared to untreated soil. Of the 22 pathways which were found to be differentially represented between the treatments, all but one had a higher relative abundance in Luna Tranquility treated soil relative to UTG. 2-nitrobenzoate degradation I pathway involved in microbial metabolism of nitrobenzoates compounds found in many pesticides [29], was upregulated by Luna Tranquility application. Another pathway overrepresented in Luna Tranquility treated soils, PWY-6210, is involved in degradation of 2-aminophenols, common components of pesticides [30]. The fact that these two pathways governing the degradation of nitrogenous pesticide-associated compounds were increased in relative abundances in soils treated with Luna Tranquility suggests that bacterial bioremediation may be a response to the treatment. In a similar manner, our previous study found that the fungicides applications were associated with an increased relative abundance of enzymes and pathways which degraded halo-organic and xenobiotic compounds [10]. One potential consequence of this bioremediation may be a reduced efficacy of the pesticide. If bacteria are increasing the speed with which the active ingredients are degraded, the span of time during which the plant is protected from pathogens may be reduced [31]. It is thus possible that Luna Tranquility may have a reduced window of protection relative to the other fungicides examined. Previously it was reported that direct soil application of fluopyram stimulated phosphorous-solubilizing bacteria [32]. Our study did not find a corresponding change to phosphorous-solubilizing function in the soil after applications of Luna Tranquility, suggesting that other environmental factors, as well as significantly reduced fungicide–soil contact, may drastically alter the effect of fungicide on the soil microbiome and its functions.

### 4.3. Plant Outcomes

The failure of any of the fungicide treatments to distinguish themselves in terms of disease prevention may be attributed to a generally low disease prevalence throughout the dataset on the whole. Only 27 out the 539 stems sampled presented with *Monilinia*, and only one showed signs of *Botrytis*. No significant differences were found the number of ripe fruits per stem at harvest time between the treatments. However, significant differences in floral development were found at the early July sampling date. The Miravis Prime treated plants had a significant delayed in floral development, while no treatment offered significantly advanced floral development at this stage relative to untreated plants. Given that no significant variance was found in either the number of ripe berries or the number of unmarketable berries between the treatments, these significant delays in floral development in Miravis Prime treated plants relative to untreated plants may not have a deleterious effect on harvest outcomes. Luna Tranquility application did not have a significant effect on plant development. Furthermore, the fungicides associated with significant effects on floral development, did not have a significant effect on soil microbiome. Results such as these suggest that fungicide-associated changes in plant development are not a result of changes to the soil microbiome. However, given that these plant development observations and soil samples were taken from plants the same year as the fungicide application, they do not necessarily indicate that the application of these fungicides will have no effect on crop outcomes or soil health in the long term. One additional qualification to the findings of our research is that the amplicon sequencing method can only shed light on the relative abundances of microbial taxa and functions, not total abundances. It is thus possible that the total microbial load of the soils may have varied significantly between the fungicides and control groups, and that one or more of the treatments in this trial may lead to changes in soil health as a result of changes to the overall microbial population. For the above reason, more research on the ecotoxicology of these fungicides is necessary to obtain a more complete idea of how they effect soil health. 

## 5. Conclusions

It is well-established that plants are supported by a wide array of beneficial fungi and bacteria. These organisms should be seen as a critical component of crop and soil health and understanding them and the ways in which they interact with agricultural practices may prove to be a key component of preserving agricultural resources. While the fungicides examined in this trial, taken as a whole, reduced bacterial diversity, one fungicide—Luna Tranquility—appeared to elicit the greatest number of changes to both the bacterial and fungal microbiome. Luna Tranquility led to significant changes in the composition of the bacterial and fungal communities relative to untreated soil, and also led to significant changes in bacterial function. Though these changes are possibly concerning, Luna Tranquility did not appear to effect in a significant way the crop outcomes analysed in this study, while three other fungicides did, despite not generating significant microbiome effects. The ambiguities presented in these findings suggest a complicated relationship between the microbial ecosystem and the crop that they support. Further research on the interactions between this system and pesticides is needed to attain a more complete idea of how best to safeguard soil health without sacrificing the agricultural productivity that agrichemicals allow. Ultimately, the results of this study have expanded the understanding of the interactions between a broad array of fungicides and the microbiome of lowbush blueberries. Additionally, it identified a possible synergetic effect of two fungistatic compounds leading to significant alterations to the soil ecosystem. 

## Figures and Tables

**Table 1:** List of fungicides used in this trial.

Trade Name	Active Ingredients	Application Rate	Mode of Action
Cabrio	pyraclostrobin	1.95 g/100 m^2^	inhibits mitochondrial cytochrome bc-1 complex
Miravis Bold	pydiflumetofen	0.84 mL/100 m^2^	inhibits succinate dehydrogenase
Sercadis	fluxapyroxad	1.30 mL/100 m^2^	inhibits succinate dehydrogenase
Scala	pyrimethanil	11.25 mL/100 m^2^	inhibits methionine production
Scholar	fludioxonil	2.04 mL/100 m^2^	inhibits a catalytic enzyme that breaks down methylglyoxal
Velum Prime	fluopyram	3.0 mL/100 m^2^	inhibits succinate dehydrogenase
Luna Tranquility	fluopyram + pyrimethanil	12 mL/100 m^2^	inhibits succinate dehydrogenase + inhibits methionine production
Miravis Prime	pydiflumetofen + fludioxonil	8.77 mL/ 100 m^2^	inhibits a catalytic enzyme that breaks down methylglyoxal + inhibits succinate dehydrogenase
Merivon	fluxapyroxad + pyraclostrobin	7.31 mL/100 m^2^	inhibits succinate dehydrogenase + inhibits mitochondrial cytochrome bc-1 complex

**Table 2:** Differing floral bud development stages by treatment group. Differing letters indicate significant differences in floral development stage at sampling time (Fisher’s LSD test, *p* < 0.05).

Treatment	Mean Floral Node Development Stage (n = 28)	Standard Deviation	Fisher’s LSD ^1^ (*p* < 0.05)
UTG	3.86	0.76	AC
Miravis Prime	3.46	0.51	B
Cabrio	3.61	0.74	BC
Scala	3.61	0.69	BC
Miravis Bold	3.68	0.61	ABC
Sercadis	3.79	0.42	AC
Luna Tranquility	3.79	0.5	AC
Scholar	3.86	0.52	AC
Velum Prime	3.96	0.74	A
Merivon	3.96	0.43	C

^1^ Significance of variance between floral development tested by Fisher’s Least Significant Difference test by treatment group. For each treatment, those with differing letters in the fourth column indicate significant difference (*p* < 0.05).

## Data Availability

The data presented in this study are openly available in NCBI sequence read archive under the accession numbers.

## Supplementary Materials

The following supporting information can be downloaded at: https://www.mdpi.com/article/10.3390/microorganisms11020410/s1, Figure S1: PCoA of fungal community structures; Figure S2: PCoA of fungal community structures.
