# Supplementary material for: Changes to Soil Microbiome Resulting from Synergetic Effects of Fungistatic Compounds Pyrimethanil and Fluopyram in Lowbush Blueberry Agriculture, with Nine Fungicide Products Tested
Source: Microorganisms. 2023 Feb 6;11(2):410. doi: 10.3390/microorganisms11020410 (PMC9968141; doi:10.3390/microorganisms11020410)
Supplement: Supplementary file 1 [file microorganisms-11-00410-s001.zip › microorganisms-2019980-supplementary.pdf]

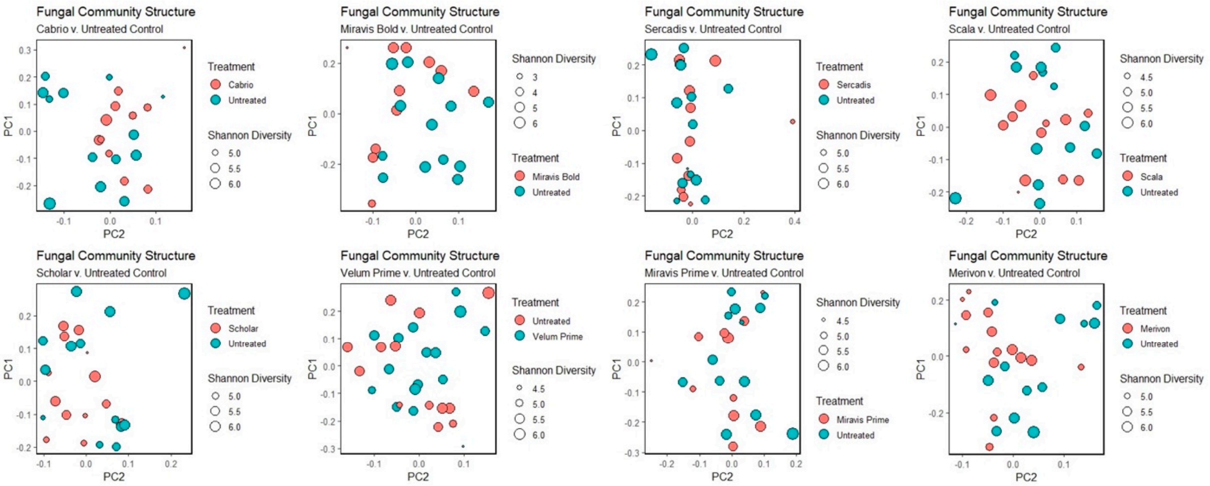

Fig. S1: PCoA of fungal community structures. PCoA comparing individual treatments and untreated control.

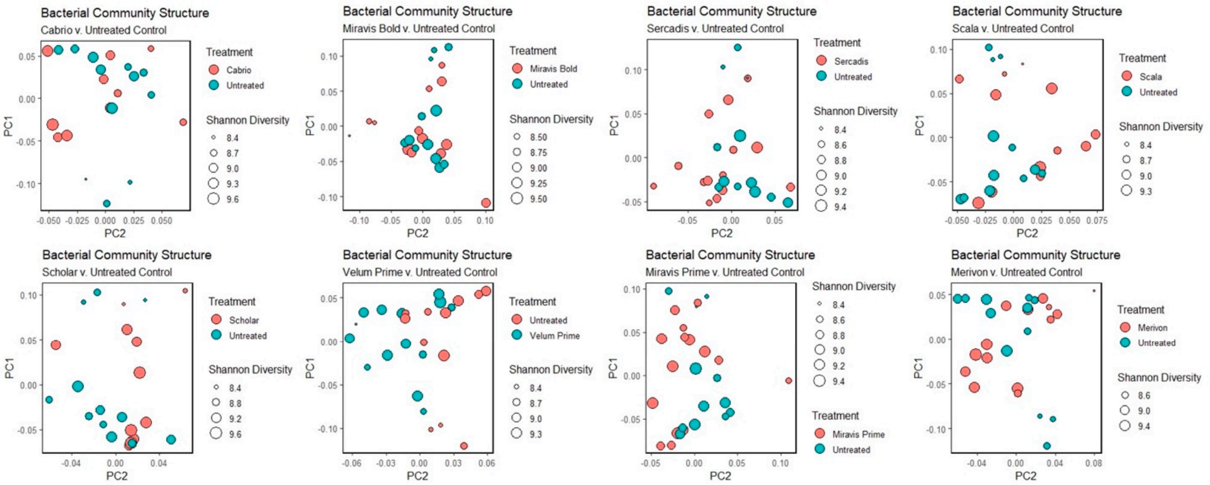

Fig. S2: PCoA of bacterial community structures. PCoA comparing individual treatments and untreated control.
